# Supplementary material for: The frequency of complications in a cohort of patients diagnosed with hemophilia A and hemophilia B receiving prophylactic treatment in Colombia: A retrospective noninterventional study
Source: PLoS One. 2023 Nov 27;18(11):e0286187. doi: 10.1371/journal.pone.0286187 (PMC10681305; doi:10.1371/journal.pone.0286187)
Supplement: Attachment — Submitted filename: PONE-D-22-11504_R.pdf [file pone.0286187.s001.pdf]

PLOS ONE

Frequency of complications in a cohort of patients diagnosed with hemophilia A or hemophilia B receiving prophylactic treatment at a health care institution in Colombia: Retrospective noninterventional study

--Manuscript Draft--

**Manuscript Number:**	PONE-D-22-11504
**Article Type:**	Research Article
**Full Title:**	Frequency of complications in a cohort of patients diagnosed with hemophilia A or hemophilia B receiving prophylactic treatment at a health care institution in Colombia: Retrospective noninterventional study
**Short Title:**	Frequency of complications of patients with hemophilia
**Corresponding Author:**	Jorge Enrique Machado-Alba, M.D; Ph.D<br>Universidad Tecnológica de Pereira<br>Pereira, Risaralda COLOMBIA
**Keywords:**	Outcome Assessment, Health Care; Hemophilia A; Hemophilia B; Factor VIII, Factor IX.
**Abstract:**	Introduction: Hemophilia A and B are disorders associated with the deficit of coagulation factors VIII and IX. Objective: was to determine the incidence of complications in a cohort of patients diagnosed with moderate and severe hemophilia A or B under treatment in a specialized institution. Methods: A retrospective study of a cohort of patients with replacement therapy for hemophilia A or B, evaluating treatment and complications between January/2012 and July/2019. The characteristics associated to disease management, sociodemographic and clinical variables were extracted from the medical records. Time to inhibitor development and rate associated to bleeding and hospitalizations were evaluated. Results: A total of 159 male patients were identified with hemophilia A (n=140; 88.1%) and B (n=19; 11.9%) with a mean follow-up of 5.9±2.3 years. The mean age was 23.6±16.1 years, hemophilia was reported as severe in 125 patients in hemophilia A (89.3%) and 13 patients in hemophilia B (68.4%). Primary prophylaxis was registered in 17.0% of patients, 44.7% secondary, and 38.3% tertiary, mainly with recombinant factors (n=84; 52.8%) followed by plasma derived factors (n=75; 47.2%). The incidence of inhibitor development was 0.3 per 100 patients/year, with mean time to event of 509 days. The incidence of bleeding was 192 per 100 patients/year, especially at the joint (n=99; 62.3%) and muscle (n=25; 15.7%) level. The incidence of hospitalization was 3.7 per 100 patients/year. Conclusions: The most common complication was joint bleeding which was expected in this type of patients. Low proportion of patients developed factor inhibitors during the follow up.
**Order of Authors:**	Jorge Enrique Machado-Alba, M.D; Ph.D
	Juan David Wilches-Gutierrez, MD, MSc
	Diana Rocio Arias-Osorio, MD
	Juan Manuel Reyes, QF, MSc
	Maria Lourdes Nakandakari, MD
	Harrison David Ospina-Arzuaga, MD
	Andres Gaviria-Mendoza, MD
	Natalia Castaño-Gamboa, Economist
	Luis Fernando Valladales-Restrepo, MD, MSc
	Manuel Enrique Machado-Duque, MD, MSc
**Additional Information:**	
**Question**	**Response**

Powered by Editorial Manager® and ProduXion Manager® from Aries Systems Corporation

**Financial Disclosure**<br><br>Enter a financial disclosure statement that describes the sources of funding for the work included in this submission. Review the submission guidelines for detailed requirements. View published research articles from *PLOS ONE* for specific examples.<br><br>This statement is required for submission and **will appear in the published article** if the submission is accepted. Please make sure it is accurate.<br><br>**Unfunded studies**<br>Enter: *The author(s) received no specific funding for this work.*<br><br>**Funded studies**<br>Enter a statement with the following details:<br>• Initials of the authors who received each award<br>• Grant numbers awarded to each author<br>• The full name of each funder<br>• URL of each funder website<br>• Did the sponsors or funders play any role in the study design, data collection and analysis, decision to publish, or preparation of the manuscript?<br>• **NO** - Include this sentence at the end of your statement: *The funders had no role in study design, data collection and analysis, decision to publish, or preparation of the manuscript.*<br>• **YES** - Specify the role(s) played.<br><br>* typeset	YES: This study was funded by Pfizer Colombia.
**Competing Interests**<br><br>Use the instructions below to enter a competing interest statement for this submission. On behalf of all authors, disclose any competing interests that could be perceived to bias this work—acknowledging all financial support and any other relevant financial or non-financial competing interests.<br><br>This statement is required for submission	yes: Reyes JM, Lourdes M and Castano N are paid employees of Pfizer Colombia. Juan David Wilches-Gutierrez, Diana Rocio Arias-Osorio are paid employees of IPS-Especializada. Andres Gaviria-Mendoza, Natalia Castaño-Gamboa, Luis Fernando Valladales-Restrepo, Manuel E. Machado-Duque and Jorge Machado-Alba are paid employees of Audifarma SA. Harrison David Ospina-Arzuaga do not have any conflict of interest.

and **will appear in the published article** if the submission is accepted. Please make sure it is accurate and that any funding sources listed in your Funding Information later in the submission form are also declared in your Financial Disclosure statement.<br><br>View published research articles from *PLOS ONE* for specific examples.<br><br>**NO authors have competing interests**<br><br>Enter: *The authors have declared that no competing interests exist.*<br><br>**Authors with competing interests**<br><br>Enter competing interest details beginning with this statement:<br><br>*I have read the journal's policy and the authors of this manuscript have the following competing interests: [insert competing interests here]*<br><br>* typeset	
**Ethics Statement**<br><br>Enter an ethics statement for this submission. This statement is required if the study involved:<br><br>• Human participants<br>• Human specimens or tissue<br>• Vertebrate animals or cephalopods<br>• Vertebrate embryos or tissues<br>• Field research<br><br>Write "N/A" if the submission does not require an ethics statement.<br><br>General guidance is provided below. Consult the submission guidelines for detailed instructions. **Make sure that all information entered here is included in the Methods section of the manuscript.**	The protocol was approved by the Bioethics Committee of the Technological University of Pereira in the category of risk-free research (endorsement code: 02-091120). The ethical principles established by the Declaration of Helsinki were respected.

**Format for specific study types**<br><br>**Human Subject Research (involving human participants and/or tissue)**<br>• Give the name of the institutional review board or ethics committee that approved the study<br>• Include the approval number and/or a statement indicating approval of this research<br>• Indicate the form of consent obtained (written/oral) or the reason that consent was not obtained (e.g. the data were analyzed anonymously)<br><br>**Animal Research (involving vertebrate animals, embryos or tissues)**<br>• Provide the name of the Institutional Animal Care and Use Committee (IACUC) or other relevant ethics board that reviewed the study protocol, and indicate whether they approved this research or granted a formal waiver of ethical approval<br>• Include an approval number if one was obtained<br>• If the study involved *non-human primates*, add *additional details* about animal welfare and steps taken to ameliorate suffering<br>• If anesthesia, euthanasia, or any kind of animal sacrifice is part of the study, include briefly which substances and/or methods were applied<br><br>**Field Research**<br><br>Include the following details if this study involves the collection of plant, animal, or other materials from a natural setting:<br>• Field permit number<br>• Name of the institution or relevant body that granted permission	
**Data Availability**<br><br>Authors are required to make all data underlying the findings described fully available, without restriction, and from the time of publication. PLOS allows rare exceptions to address legal and ethical concerns. See the PLOS Data Policy and FAQ for detailed information.	Yes - all data are fully available without restriction

Powered by Editorial Manager® and ProduXion Manager® from Aries Systems Corporation

A Data Availability Statement describing where the data can be found is required at submission. Your answers to this question constitute the Data Availability Statement and **will be published in the article**, if accepted.<br><br>**Important:** Stating 'data available on request from the author' is not sufficient. If your data are only available upon request, select 'No' for the first question and explain your exceptional situation in the text box.<br><br>Do the authors confirm that all data underlying the findings described in their manuscript are fully available without restriction?	
**Describe where the data may be found in full sentences. If you are copying our sample text, replace any instances of XXX with the appropriate details.**<br><br>• If the data are **held or will be held in a public repository**, include URLs, accession numbers or DOIs. If this information will only be available after acceptance, indicate this by ticking the box below. For example: *All XXX files are available from the XXX database (accession number(s) XXX, XXX.).*<br>• If the data are all contained **within the manuscript and/or Supporting Information files**, enter the following: *All relevant data are within the manuscript and its Supporting Information files.*<br>• If neither of these applies but you are able to provide **details of access elsewhere**, with or without limitations, please do so. For example:<br><br>*Data cannot be shared publicly because of [XXX]. Data are available from the XXX Institutional Data Access / Ethics Committee (contact via XXX) for researchers who meet the criteria for access to confidential data.*<br><br>*The data underlying the results presented in the study are available from (include the name of the third party*	Data availability: protocols.io<br>Data access: dx.doi.org/10.17504/protocols.io.b4hwqt7e

and contact information or URL).<br>• This text is appropriate if the data are owned by a third party and authors do not have permission to share the data.<br><br>* typeset	
Additional data availability information:	

Manuscript Click here to access/download;Manuscript;Haemophilia (PO).docx

Original article

Frequency of complications in a cohort of patients diagnosed with hemophilia A or hemophilia B receiving prophylactic treatment at a health care institution in Colombia. Retrospective noninterventional study

Running title: Frequency of complications of patients with hemophilia

Authors

Jorge E Machado Alba [1(ORCID: 0000-0002-8455-0936)], Juan David Wilches-Gutierrez[2], Diana Rocio Arias-Osorio[2], Juan Manuel Reyes [3(ORCID: 0000-0003-0806-7173)], Maria Lourdes Nakandakari [3], Harrison David Ospina-Arzuaga [1], Andres Gaviria-Mendoza [1,4(ORCID: 0000-0003-2500-7658)], Natalia Castaño-Gamboa [3(ORCID: 0000-0001-7383-6800)], Luis Fernando Valladales-Restrepo [1,4(ORCID: 0000-0002-4245-0101)], Manuel E. Machado-Duque [1,4(ORCID: 0000-0001-8458-0986)].

[1]Grupo de Investigación en Farmacoepidemiología y Farmacovigilancia. Universidad Tecnológica de Pereira-Audifarma S.A
[2]IPS Especializada. Bogotá, Colombia.
[3]Pfizer Colombia. Bogotá, Colombia.
[4]Grupo de Investigación en Biomedicina, Fundación Universitaria Autónoma de las Américas, Pereira, Colombia.

Correspondence: Jorge Enrique Machado-Alba. Grupo de Investigación en Farmacoepidemiología y Farmacovigilancia. Universidad Tecnologica de Pereira-Audifarma S.A, Address: Calle 105 No. 14-140, Pereira, Risaralda, Colombia, Zip Code: 660003. E-Mail: machado@utp.edu.co phone: 57 3108326970; fax: 57 63137822.

Author mails: juan.wilches@ipsespecializada.com.co; diana.arias@ipsespecializada.com.co; juanmanuel.reyes@pfizer.com; maria.nakandakari@pfizer.com; hdoa74@utp.edu.co; angaviria@utp.edu.co; NATALIA.CASTANO@pfizer.com; lfvalladales@utp.edu.co; memachado@utp.edu.co

Word count abstract: 249

Word count main text: 3002

Number of tables: 4

Number of figures: 0

Number of references: 21

ABSTRACT

Introduction: Hemophilia A and B are disorders associated with the deficit of coagulation factors VIII and IX. *Objective*: was to determine the incidence of complications in a cohort of patients diagnosed with moderate and severe hemophilia A or B under treatment in a specialized institution. *Methods*: A retrospective study of a cohort of patients with replacement therapy for hemophilia A or B, evaluating treatment and complications between January/2012 and July/2019. The characteristics associated to disease management, sociodemographic and clinical variables were extracted from the medical records. Time to inhibitor development and rate associated to bleeding and hospitalizations were evaluated. *Results*: A total of 159 male patients were identified with hemophilia A (n=140; 88.1%) and B (n=19; 11.9%) with a mean follow-up of 5.9±2.3 years. The mean age was 23.6±16.1 years, hemophilia was reported as severe in 125 patients in hemophilia A (89.3%) and 13 patients in hemophilia B (68.4%). Primary prophylaxis was registered in 17.0% of patients, 44.7% secondary, and 38.3% tertiary, mainly with recombinant factors (n=84; 52.8%) followed by plasma derived factors (n=75; 47.2%). The incidence of inhibitor development was 0.3 per 100 patients/year, with mean time to event of 509 days. The incidence of bleeding was 192 per 100 patients/year, especially at the joint (n=99; 62.3%) and muscle (n=25; 15.7%) level. The incidence of hospitalization was 3.7 per 100 patients/year. *Conclusions*: The most common complication was joint bleeding which was expected in this type of patients. Low proportion of patients developed factor inhibitors during the follow up.

Keywords: Outcome Assessment, Health Care; Hemophilia A; Hemophilia B; Factor VIII, Factor IX.

Introduction

Hemophilia A and hemophilia B are coagulation disorders related to the deficiency of factors VIII and IX, respectively. In Colombia, they are considered rare or orphan d[illegible]ses, since their prevalence is approximately 1 case per 5,000 people [1, 2]. Patients with severe hemophilia are those with less than 1 IU/dL of the deficient factor [3, 4]; patients with levels between 1 and 5 IU/dL and between 5 IU/dL and 40 IU/dL are classified as moderate and mild, respectively [4]. The most severe forms of hemophilia are characterized by spontaneous bleeding in the joints and muscles [3]. Repeated bleeding in the same locations can lead to hemophilic arthropathy [5].

Quality care for these conditions, especially severe cases, requires the intervention of a multidisciplinary team focused on the prevention of bleeding and joint damage, the management of bleeding episodes, rehabilitation, pain management, psychosocial intervention and education [4]. Through such efforts, joint damage is prevented or delayed, the patient's quality of life is preserved [6], and risk factors that negatively modify the evolution of the condition are mitigated [4]. In severe cases, prophylaxis serves as a pharmacological strategy focused on the prevention of bleeding, which can be primary, secondary or tertiary depending on the history of bleeding and joint damage [4, 7].

The complications related to treatment with antihemophilic factors that has the greatest impact is the development of inhibitors, since they affect the effectiveness of coagulation factors and increase the risks of morbidity and mortality and the costs to the health system [3, 4, 8, 9]. Real-world studies allow the evaluation of the clinical outcomes of patients under routine conditions. These findings provide decision-makers with information related to the effectiveness and safety of pharmacological technologies and consider scenarios that cannot always be evaluated in clinical studies. Additionally, they can complement available information to provide data on how doctors and patients choose

treatments, dosages and regimens and on the frequency of use of health services and technologies and their effectiveness [5, 10].

Currently, in Colombia, there are no studies regarding the follow-up, treatment and outcomes of patients with hemophilia A and B; therefore, it is important to expand knowledge regarding this population. This need has led to the objective of evaluating the incidence of adverse outcomes in a cohort of patients diagnosed with hemophilia A and hemophilia B at a health care provider (Institución Prestadora de Servicios Especializada: IPS-E) of Audifarma SA) in Colombia.

Materials and methods

This was a retrospective study of a dynamic cohort of patients diagnosed with hemophilia A and hemophilia B who entered the Coagulopathies Program of IPS-E for replacement therapy between January 2012 and July 2019. Given the descriptive nature of the study, the evolution of the treatment and the patients' clinical results were evaluated during the follow-up period. The index date was defined as the date of admission to the IPS-E Coagulopathies Program and the initiation of some recombinant or plasma-derived antihemophilic factor within a prophylaxis regimen. The follow-up time began from the index date to inhibitors development, ≥0.6 Bethesda units/milliliters (BU/ml), death, loss to follow-up, abandonment of the Program or July 2019, whichever came first.

Inclusion criteria were male patients diagnosed with hemophilia A or B, who had been registered with the Specialized Coagulopathies Program of IPS-E for 6 or more months and were undergoing prophylactic treatment with recombinant or plasma-derived factors. Those who presented inhibitors upon entering the program or who were participating in a clinical trial were excluded. Sample size

estimation was not performed due to the design of the study; therefore, all of the patients in the Program who met the inclusion criteria were included in the cohort.

All clinical, treatment and outcome information were obtained from the patients' medical records, which were reviewed by two trained physicians, and all information was recorded in an electronic collection format. The following groups of variables were collected:

a) *Sociodemographic*: age, sex, city of residence, weight, height, family history of hemophilia and development of inhibitors.

b) *Clinical*: type of hemophilia, date of diagnosis, presence of arthropathy, history of bleeding and previous treatment.

c) *Pharmacological:* type of prophylaxis (primary, secondary, tertiary), type of factor, dose, administration interval, switches in coagulation factors, interruptions, and their causes.

d) *Adverse events:* bleeding, site of bleeding, trauma, surgeries, hospitalizations, development of inhibitors (BU), anaphylactic reactions or nephrotic syndrome. Finally, patients who developed inhibitors were followed.

The main outcomes were the development of inhibitors, severity of bleeding, trauma, hospitalizations, and anaphylactic reactions. These outcomes were determined by the information available in the medical records. Additionally, data related to treatments performed in the 6 months prior to admission to the Specialized HCP Coagulopathies Program were taken into account.

Statistical analysis

Descriptive data analysis

The database was created in Microsoft Excel 2020 for Windows. Analyses were performed using the statistical package SPSS 28.0 (IBM, New York, NY, USA). The information was validated to ensure that the variables had been adequately defined and the data is complete. Dichotomous and categorical variables are described as percentages. For continuous variables, the means, medians, interquartile ranges (IQR) and standard deviations (SD) were estimated. Additionally, the normality of continuous variables was analyzed using distribution graphs and the Kolmogorov–Smirnov test. For the analysis of the time to the presentation of the event, the survival function was developed using the Kaplan–Meier method. Patients who discontinued the prophylaxis and those who were lost to follow-up were censored.

Inference analysis

For the analysis of continuous variables with normal behavior, Student's t test was used, and for variables with nonnormal behavior, the Mann–Whitney test was used. For categorical variables, X^2 tests and Fisher's test were performed. A p-value <0.05 was established as statistically significant.

Results

A total of 207 patients undergoing prophylactic treatment were identified. Of these, 40 already had inhibitors before entering the program, five who were not in the program for at least 6 months, two who did not have a complete medical record, and one who was participating in a clinical trial were

excluded. Finally, 159 subjects met the inclusion criteria. They were followed for an average of 5.9±2.3 years and had an average age at admission of 23.6±16.1 years. The majority of patients were classified in the severe hemophilia A group. The patients were distributed mainly in the cities of Bogotá/Cundinamarca (n=87; 54.7%), Cali/Valle del Cauca (n=28; 17.6%) and Medellín/Antioquia (n=10; 6.3%), followed by 11 other departments in Colombia (n=34; 21.4%). Fifty-three 53 patients had some family history of hemophilia (Table 1).

Table 1. Demographics and clinical characteristics prior to follow-up in 159 patients with hemophilia A and B on prophylaxis in Colombia, 2012-2019.

Characteristics	Frequency	%
**Hemophilia A**		
Severe	125	89.3
Moderate	15	10.7
**Hemophilia B**		
Severe	13	68.4
Moderate	6	31.6
**History of hemophilia**		
Family history of hemophilia	53	33.3
Family history of inhibitors	2	1.3
**Age at diagnosis**		
0 - 12 months	89	56
12 - 60 months	33	20.8
> 60 months	2	1.2
No data available	35	22
**Previous complications**		
Arthropathy	126	79.2
Number of joints with arthropathy (median, IQR)	2	1 - 4
Bleeding 6 months prior	19	11.9
Number of bleeds (median, IQR)	1	1 - 1
**Previous treatments**		
Plasma derived factor IX	12	7.5
Recombinant factor IX	6	3.8
Plasma derived factor VIII	62	39
Recombinant factor VIII	57	35.8
No previous treatment	8	5
No data available	14	8.8
**Previous weekly factor dose**		
Dose (IU per week) †	4500	2500 - 5000
On demand	2	1.3

Number applications per week	3	2 - 3

† Patients with data recorded in clinical records. **IU:** international units. **IQR:** interquartile range

In the cohort, 47 patients (29.6%) had some associated comorbidity, the most frequent were arterial hypertension (n=12; 7.5%), non-ulcer dyspepsia (n=3; 1.9%), type 2 diabetes mellitus (n=1; 0.6%) and chronic obstructive pulmonary disease (COPD) (n=1; 0.6%). However, they also had other infections, such as hepatitis C virus (HCV) infection (n=24; 15.1%), human immunodeficiency virus (HIV) (n=2; 1.2%) and hepatitis B virus (HBV) (n=2; 1.2%). The patients had infectious comorbidities, the mean was more than 10 years in total at the time of admission to the Program and had an average age of 39.9 years.

Prior to admission to the cohort, 121 (76.1%) patients were receiving prophylaxis (Previously Treated Patients – PTPs), and none of these had low or high response inhibitors. Table 1 shows the proportions of patients according to the type of hemophilia, severity, age at the time of diagnosis, family history, complications prior to admission (especially arthropathy) and previous management.

The most frequent type of management was secondary prophylaxis (n=71; 44.7%), followed by tertiary prophylaxis (see Table 2). The most frequently used coagulation factor was recombinant (n=84, 52.8% of all patients); in 68 patients (80.9%), there was no clinical record of the reason for its use, while in 8 (9.5%) cases, the reported reason was previous hypersensitivity to the plasma derivative. Table 2 also shows the distribution of patients according to the type of prophylaxis, the factors used, the weekly doses of antihemophilic factor and other reasons for switches or modifications of treatment (in 42 cases, 26.4%) during the follow-up period, in addition to the frequency and causes of discontinuation/interruption of treatment (36 cases, 22.6%), with loss of affiliation with the insurance health company (Entidad Promotora de Salud- EPS) being the most

frequent (n=11; 6.9%).). The median time of discontinuation of treatment was 3 months, mainly due to loss of affiliation and administrative problems with the EPS.

Table 2. Practice patterns among the study cohort regarding treatment regimen, product type, prescribe[illegible]ctor dosing, switch, discontinuation, and interruption in 159 patients with hemophilia A and B on prophylaxis in Colombia.

Treatment characteristic	Frequency	%
**Type of prophylaxis**		
Primary	27	17.0
Secondary	71	44.7
Tertiary	61	38.4
**Treatments**		
Plasma derived factor IX	11	6.9
Recombinant factor IX	8	5
Plasma derived factor VIII	62	39
Recombinant factor VIII/ von Willebrand factor	1	0.6
Recombinant factor VIII	77	48.4
**Weekly factor dose**		
Dose (IU per week) †	4500	(2500 - 5000)
Number applications per week	3	(2 - 3)
Initial treatment duration (months) (median, IQR)	66	(21-88)
Use of recombinant factors	114	71.7
**Reason for use of recombinant factor**		
Prior hypersensitivity to plasma derivatives	8	9.5
Poor response to plasma derivatives	3	3.6
Shortage of plasma derivatives	3	3.6
Clinical preference due to age and risk of infection	1	1.2
Patient request	1	1.2
No data available	68	80.9
**Switch of treatment**		
From recombinant factor to plasma derivative	1	0.6
From plasma derivative to recombinant facto[illegible]	27	17.0
From recombinant factor to recombinant factor	3	1.9
From plasma derivate to plasma derivate	9	5.7
**Discontinuation**		
Discontinuation of prophylaxis (at least one week)	36	22.6
Time without receiving prophylaxis (months) (median; IQR)	3	(1 - 7)
**Causes of interruption/discontinuation**		
Loss of EPS affiliation	11	6.9
Administrative problem with EPS	10	6.3
Poor adherence/not attending to follow-ups	6	3.8
Patient's decision	3	1.9
Transfer to area without coverage	2	1.3
Medical decision (improvement in bleeding frequency)	1	0.6
Anaphylactic reaction	1	0.6
No data available	2	1.3

† **Patients with data recorded in clinical records.** EPS: **Insurance Health Company (Entidad Promotora de Salud).** IU: **international units.** IQR: **interquartile range.**

Twenty-eight (17.6%) switched therapy were identified in all patients, the majority of the switches between the different coagulation factors used was the transition from plasma derivatives to recombinant treatment in both the hemophilia A and hemophilia B patients. Table 3 shows the frequency of switches between the types of coagulation factors used.

Table 3. Number patients who switched treatment regimen during the follow up according to the treatment regimen, in 159 patients with hemophilia A and B on prophylaxis in Colombia.

	Total of patients	Non switchers<br>n (%)	Switch to recombinant factor IX<br>n (%)	Switch to recombinant factor VIII<br>n (%)	Switch to plasma derived factor VIII<br>n (%)
**Hemophilia A**					
Plasma derived factor VIII	62 (44.3%)	40 (64.5%)	NA	22 (35.5%)	0 (0.0)
Recombinant factor VIII	77 (55.0%)	76 (98.7%)	NA	0 (0.0)	1 (0.3%)
Recombinant factor VIII/ von Willebrand factor	1 (0.7%)	1 (100.0%)	NA	0 (0.0)	0 (0.0)
**Hemophilia B**					
Plasma derived factor IX	11 (57.9%)	6 (54.5%)	5 (45.5%)	NA	NA
Recombinant factor IX	8 (42.1%)	8 (100.0%)	0 (0.0%)	NA	NA

NA: not applicable

Regarding complications and events in the patients with hemophilia who were receiving prophylaxis, the vast majority of the patients had suffered some type of trauma, almost one-third had undergone ~~some type of~~ surgical intervention, and almost one-fifth had to be hospitalized during the follow-up period. More than 90% had had some type of hemorrhage, most commonly articular and of traumatic origin; of these patients, the majority required the administration of antihemophilic factors. Only 2 (1.2%) patients had experienced anaphylactic reactions (~~see~~ Table 4).

Table 4. Total number of traumas, surgeries, hospitalization, inhibitors, and bleeding events in 159 patients with hemophilia A and B on prophylaxis in Colombia.

	Frequency	%	Hemophilia A	%	Hemophilia B	%
**Bleedings**						
Number of patients	159		140		19	

Patients with bleed during follow-up	144	90.6	128	91.4	16	84.2
Total frequency of patients during follow-up (median; IQR)	11.3	12.2	11.4	12.6	10.6	9.1
Total of follow-up	7	(5 - 8)	6.8	(4.8 - 7.7)	7.6	(6.9 - 7.7)
Mean (# patient bleeds / year)	20.5		18.8		2.1	
Type of first bleeding						
Spontaneous	31	21.5	25	19.5	6	37.5
Joint	15	10.4	15	11.7	3	18.8
No joint	16	11.1	10	7.8	3	18.8
Traumatic	97	67.4	88	68.8	9	56.3
Joint	71	49.3	66	51.6	5	31.3
No joint	26	18.1	22	17.2	4	25.0
Not classified	16	11.1	15	11.7	1	6.3
Number of bleeding with factor requirement	144	100	128	100	16	100
**Trauma**						
Number of patients	137	86.2	121	86.4	16	84.2
Frequency (median; IQR)	5	(2 - 11)	5	(1.2 - 11.7)	5	(2 - 10)
Incidence (100 patients / year)	143		177.9		21.1	
Site						
Joint	273	74.0	246	75.9	27	60.0
Head	17	4.6	16	4.9	1	2.2
Muscular	70	19.0	54	16.7	16	35.6
ENT	9	2.4	8	2.5	1	2.2
**Surgery**						
Number of patients	47	29.6	38	27.1	9	47.4
Frequency (median; IQR)	0	(0 - 1)	0	(0 - 1)	0	(0 - 0)
Incidence (100 patients / year)	5.9		2.5		9.0	
Type						
Major	20	12.6	15	10.7	5	26.3
Minor	36	22.6	29	20.7	7	36.8
More frequent surgeries						
Tooth extraction	18	32.1	15	33.3	3	25.0
Hip replacement	6	10.7	4	8.9	3	25.0
Knee replacement	3	5.4	3	6.7	0	0.0
Knee arthroscopy	2	3.6	2	4.4	0	0.0
Other	27	48.2	21	46.7	6	50.0
**Hospitalizations**						
Number of patients	27	17	25	17.9	2	10.5
Frequency (median; IQR)	0	(0 - 1)	0	(0 - 1)	0	(0 - 1)
Incidence (100 patients / year)	3.7		3.7		0.3	
Cause						
Bruising	11	31.4	11	33.3	0	0.0
Hemarthrosis	5	14.3	5	15.2	0	0.0
Other bleedings	4	11.4	2	6.1	2	11.8
Other causes	15	42.9	15	45.5	15	88.2
**High dose of factor**						
Hip replacement surgery patient	1	0.6	1		0	0
**Inhibitors**						
Number of patients	3	1.9	3	2.1	0	0
Low response	2	1.3	2	1.4	0	0
High response	1	0.6	1	0.7	0	0
Incidence (100 patients / year)	0.3		0.4		0	
Time of inhibitor development (mean; SD)	509	677	509	677	NA	NA

**Anaphylactic reactions or nephrotic syndrome**						
Anaphylactic reactions plasma derived factor IX	1	0.6	1	0.7	0	0
Anaphylactic reactions recombinant factor IX	1	0.6	1	0.7	0	0
Nephrotic syndrome	1	0.6	1	0.7	0	0

ENT:Ear-Nose-Throat**. . IQR:** interquartile range. **SD**: standard deviation

Bivariate analysis

Bleeding

In determining the association of the variables with the presence of bleeding, statistical significance was found for arterial hypertension (OR:0.162; 95%CI:0.045-0.623; p=0.016), and those from the department of Cauca (OR:0.114; 95%CI:0.023-0.571; p=0.019) had a lower probability of experiencing hemorrhage. ~~The rest of the variables did not present any significant associations~~.

Hospitalizations

None of the examined variables were significantly associated with the need for hospitalization in patients with hemophilia undergoing prophylactic management.

Development of inhibitors

The bivariate analysis found that patients with moderate hemophilia had a higher risk of developing inhibitors against coagulation factors (OR:14.421; 95%CI:1.247-166.772; p=0.046). The three patients who developed inhibitors (two with moderate hemophilia and one with severe hemophilia) during the observation period underwent additional follow-up. Two of these patients had low response inhibitors that became negative during the observation period; they did not require switches in the prophylactic medication used, but the dose was increased to 500 IU three times a week

(previously 250 IU/three times a week (mean 27.7 IU/kg/dose in those patients). One patient with high response inhibitors (35.9 BU) was identified; this patient was a 2-year-old infant with severe hemophilia A. The patient showed continued immunotolerance of recombinant factor VIII (250 IU 1 time per week); factor VIIa (Novoseven® 1 mg/3 times week) was added, followed in February 2018 by the addition of human plasma protein with activity against factor VIII inhibitors at a dose of 1000 IU 3 times a week. This regimen was continued for 22 months until the patient achieved negative inhibitors in January 2020. A slight increase to 1.1 BU was observed at the last evaluation (April 2020). It should be noted that this same patient presented 21 bleeds due to traumatic causes with factor requirements from the diagnosis of inhibitors to the last reporting date.

Discussion

Based on the information for the cohort of patients with hemophilia A and B receiving prophylactic management at a IPS-E, it was possible to identify the sociodemographic and clinical characteristics and clinical outcomes of interest, such as the frequency of bleeding, trauma and inhibitor development during follow-up, in addition to the drugs used, their doses and any switches. The evaluation of these results allows the continuous improvement of the health system [10, 11]. Additionally, the information collected could contribute to the development of mechanisms to guide therapeutic strategies, access to pharmacological treatments and the generation of knowledge based on real-world evidence of patients with hemophilia in Colombia.

Within the selected cohort of patients with severe hemophilia, more than half (56%) were diagnosed in the first 12 months of life, and the remaining cases for whom this data were available were diagnosed between 12 and 60 months of age (22%); this finding is consistent with the reports of epidemiological studies in which patients with severe forms of the disease were diagnosed during the

first years of life [3, 12]. This behavior may be related to the adoption of a regulatory framework that promotes comprehensive and timely access to orphan diseases [2]. In addition, patients who entered the follow-up cohort during the observation period had an average age of approximately 24 years, which implies a long evolution of the disease and complications prior to admission to the Coagulopathies Program, as is evident in the study. A high proportion of patients with arthropathy at admission (approximately 80%) had two affected joints, a situation to which clinicians responsible for their care and attention should be alert given its impact on the burden of the disease, quality of life and the need for tertiary prevention. Studies conducted in Austria with patients with severe hemophilia show comparable proportions of arthropathy (76.7%) [10], a complication that can be reduced in these patients with adequate prophylaxis, as reported by Manco-Johnson et al. [11] and as recommended by the World Federation of Hemophilia (2).

The standard of treatment for severe cases is the regular replacement of factor through prophylaxis with antihemophilic factors or other hemostatic products to prevent bleeding; it is recommended that such prophylaxis be started at an early age to avoid musculoskeletal complications [4, 13]. However, the present study shows that only 17% of the cohort were receiving primary prophylaxis; this explains the high number of patients who had complications prior to inclusion in the cohort, as many of them were receiving secondary and tertiary prophylaxis [14]. This situation is consistent with studies conducted in the United States showing that between 1999 and 2010, the proportion of patients receiving prophylaxis increased from 40% to 80%, which had an impact on the reduction of new bleeding [11].

In the present study, the frequency of recombinant factor use increased from 36% prior to admission to the cohort to 47.8%, a finding that was associated with hypersensitivity and inadequate response to previous therapies. The most frequent change was from plasma derivatives to recombinants. Similar findings were reported in other studies, such as that of Ay et al., which reported that as of

~~2020,~~ up to 74.5% of patients with severe forms of the disease were using recombinants [10] and indicated that 3 administrations per week of factor VIII was the most frequent interval [10, 11].

The interruption or discontinuation of prophylaxis for at least one week in almost a quarter of patients throughout the follow-up period was one of the most relevant problems and was often associated with administrative problems with the EPS and loss of insurance status, a situation that can cause the patient to miss check-up visits and increases the probability of negative outcomes. The adherence report by Tencer et al. showed a median adherence of 76% in the United States [15]; additionally, Khair K et al. ~~(2008)~~, in a multicenter study in the United States, Canada, United Kingdom and Sweden, found a median adherence rate of 58.8%, and a similar study identified barriers to adherence to prophylaxis, including issues associated with health systems and others related to the patient and his family [17].

According to the patients' clinical records, 90.6% of the patients had at least one bleeding event during the follow-up period. This finding is consistent with reports of other series of patients with hemophilia A and B, which indicated that between 92.0% and 94.2.% of patients experienced a bleeding event from 1997 to 2003 [18]. However, ~~finding that~~ 9.4% of the patients did not experience bleeding with prophylaxis over an average of almost 6 years of follow-up is comparable to reports from developed countries of rates between 5.7% and 41% [5, 10]. Considering that the majority of the patients in the present study were in secondary and tertiary prophylaxis, the incidence of 19.2 bleeds per 1000 patients per year (1.9 mean bleeds per year per patient) represents a lower mean annual bleeding rate than that reported for hemophilia A patients in the United States ~~in the real-life study of~~ Yan S et al.; ~~those authors~~ reported between 2.5 and 3.9 bleeds per patient per year [19]. ~~Nonetheless, there may be~~ differences among countries related to the dose of the factor used, the type of mutation and the type of clinical follow-up, as well as patient differences in adherence and behavior [3, 10, 20].

In the evaluated group, the low frequency of development of inhibitors of coagulation factors, and even the recognition that most cases reflected a low response that later became negative, can be explained by the appropriate follow-up of the patients in this program. However, additional studies are required to confirm this association, given the low number of patients in this study who developed inhibitors. Another study reported that a history of not knowing the type of factor a patient used before entering a hemophilia treatment cohort may be associated with an increased risk of inhibitor development [21].

Some limitations should be recognized, such as the fact that data were obtained from outpatient clinical records; however, the care process was detailed in the medical records, which allowed the collection of complete and high-quality information. Other limitations include the small number of patients with severe/moderate hemophilia who met the inclusion criteria and therefore the low frequency of some outcomes, which did not allow statistical analysis. Finally, there was significant heterogeneity in the identified patients as a result of the long evolution of the disease prior to admission to the follow-up cohort, which led to a high frequency of complications and a lack of information about the follow-up and treatments that the patients had received in previous years. In addition, strengths of the study are recognized, such as the complete follow-up of each of the patients for a long period and the description of their treatments and outcomes, which resulted in the complete collection and analysis of the data, and the inclusion of a larger sample of patients than has not previously been reported in Colombia.

Conclusion

These findings show that in this cohort of patients with hemophilia A and B undergoing prophylactic management, especially those undergoing secondary and tertiary, the use of recombinant coagulation

factors led to bleeding rates similar to those reported by high-income countries and a frequency of complications comparable to the reports in the published literature, which are mainly based on the experiences of developed countries.

Declarations

Declaration of interests: Reyes JM, Lourdes M and Castano N are paid employees of Pfizer Colombia. Juan David Wilches-Gutierrez, Diana Rocio Arias-Osorio are paid employees of IPS-Especializada. Andres Gaviria-Mendoza, Natalia Castaño-Gamboa, Luis Fernando Valladales-Restrepo, Manuel E. Machado-Duque and Jorge Machado-Alba are paid employees of Audifarma SA. Harrison David Ospina-Arzuaga do not have any conflict of interest.

Funding:
This study was funded by Pfizer Colombia.

Data availability: protocols.io

Data access: dx.doi.org/10.17504/protocols.io.b4hwqt7e

Acknowledgments: not applicable.

Author contributions: JEMA participated in the drafting, data collection, data analysis, description of results, discussion, critical revision of the article, and evaluation of the final version of the manuscript. LFVR, AGM, LFVR, MEMD participated in the drafting, data collection, data analysis, description of results and discussion. HDOA participated in collect information and results. JDWG and DRAO participated in the drafting, and discussion of the article. JMR, NC and ML participated in discussion.

Ethics approval: The protocol was approved by the Bioethics Committee of the Technological University of Pereira in the category of risk-free research (endorsement code: 02-091120). The ethical principles established by the Declaration of Helsinki were respected.

References

1. Wong T, Recht M. Current options and new developments in the treatment of haemophilia. Drugs. 2011;71(3):305-20.
2. Ley 1438 de 2011 - Por medio de la cual se reforma el Sistema General de Seguridad Social en Salud y se dictan otras disposiciones. República de Colombia, Gobierno Nacional. [cited 2021 Oct 12]. Available from: https://www.minsalud.gov.co/Normatividad_Nuevo/LEY%201438%20DE%202011.pdf, (2011).
3. Berntorp E, Fischer K, Hart DP, Mancuso ME, Stephensen D, Shapiro AD, et al. Haemophilia. Nat Rev Dis Primers. 2021;7(1):45.
4. Srivastava A, Santagostino E, Dougall A, Kitchen S, Sutherland M, Pipe SW, et al. WFH Guidelines for the Management of Hemophilia, 3rd edition. Haemophilia. 2020;26(S6):1-158.
5. Berntorp E, Dolan G, Hay C, Linari S, Santagostino E, Tosetto A, et al. European retrospective study of real-life haemophilia treatment. Haemophilia. 2017;23(1):105-14.
6. Nijdam A, Kurnik K, Liesner R, Ljung R, Nolan B, Petrini P, et al. How to achieve full prophylaxis in young boys with severe haemophilia A: different regimens and their effect on early bleeding and venous access. Haemophilia. 2015;21(4):444-50.
7. Fischer K, Konkle B, Broderick C, Kessler CM. Prophylaxis in real life scenarios. Haemophilia. 2014;20 Suppl 4:106-13.
8. Blatný J, Kardos M, Miljic P, Bilić E, Benedik-Dolničar M, Faganel-Kotnik B, et al. Incidence of inhibitor development in PUPs with severe Haemophilia A in the CEE region between 2005 and 2015. Thromb Res. 2021;198:196-203.
9. Kershaw G. Detection and Measurement of Factor Inhibitors. Methods Mol Biol. 2017;1646:295-304.

10. Ay C, Perschy L, Rejtö J, Kaider A, Pabinger I. Treatment patterns and bleeding outcomes in persons with severe hemophilia A and B in a real-world setting. Ann Hematol. 2020;99(12):2763-71.

11. Manco-Johnson MJ, Soucie JM, Gill JC. Prophylaxis usage, bleeding rates, and joint outcomes of hemophilia, 1999 to 2010: a surveillance project. Blood. 2017;129(17):2368-74.

12. Hassan S, van Balen EC, Smit C, Mauser-Bunschoten EP, van Vulpen LFD, Eikenboom J, et al. Health and treatment outcomes of patients with hemophilia in the Netherlands, 1972-2019. J Thromb Haemost. 2021;19(10):2394-406.

13. Nijdam A, Altisent C, Carcao MD, Cid AR, Claeyssens-Donadel S, Kurnik K, et al. Bleeding before prophylaxis in severe hemophilia: paradigm shift over two decades. Haematologica. 2015;100(3):e84-6.

14. Fischer K, van der Bom JG, Mauser-Bunschoten EP, Roosendaal G, Prejs R, de Kleijn P, et al. The effects of postponing prophylactic treatment on long-term outcome in patients with severe hemophilia. Blood. 2002;99(7):2337-41.

15. Tencer T, Roberson C, Duncan N, Johnson K, Shapiro A. A haemophilia treatment centre-administered disease management programme in patients with bleeding disorders. Haemophilia. 2007;13(5):480-8.

16. Khair K, Lawrence K, Butler R, O'Shea E, Christie BA. Assessment of treatment practice patterns for severe hemophilia A: a global nurse perspective. Acta Haematol. 2008;119(2):115-23.

17. Thornburg CD, Duncan NA. Treatment adherence in hemophilia. Patient Prefer Adherence. 2017;11:1677-86.

18. Shih MY, Wang JD, Yin JD, Tsan YT, Chan WC. Differences in Major Bleeding Events Between Patients With Severe Hemophilia A and Hemophilia B: A Nationwide,

Population-Based Cohort Study. Clin Appl Thromb Hemost. 2019;25:1076029619888023.

19. Yan S, Maro GS, Desai V, Simpson ML. A Real-World Analysis of Commonly Prescribed FVIII Products Based on U.S. Medical Charts: Consumption and Bleeding Outcomes in Hemophilia A Patients. J Manag Care Spec Pharm. 2020;26(10):1258-65.

20. Mannucci PM, Franchini M. Is haemophilia B less severe than haemophilia A? Haemophilia. 2013;19(4):499-502.

21. Machado-Alba JE, Chica-Quintero LA, Machado-Duque ME, Gaviria-Mendoza A, Wilches-Gutierrez JD, Arias-Osorio DR. Factors Involved in the Development of Inhibitory Antibodies in Patients with Hemophilia in Colombia: A Case-Control Study. Clin Med Insights Blood Disord. 2020;13:2634853520962467.
