# Supplementary material for: The frequency of complications in a cohort of patients diagnosed with hemophilia A and hemophilia B receiving prophylactic treatment in Colombia: A retrospective noninterventional study
Source: PLoS One. 2023 Nov 27;18(11):e0286187. doi: 10.1371/journal.pone.0286187 (PMC10681305; doi:10.1371/journal.pone.0286187)
Supplement: Attachment — Submitted filename: Comments.pdf [file pone.0286187.s002.pdf]

Manuscript Title “**Frequency of complications in a cohort of patients diagnosed with hemophilia A or hemophilia B receiving prophylactic treatment at a health care institution in Colombia: Retrospective noninterventional study**”.

**General comments**

- The topic is important, however, the paper needs revision and further editing.
- All comments are mentioned on the text of the paper.

**Specific comments**

**Title:** the suggested title is **" The Frequency of complications in a cohort of patients with hemophilia A and B receiving prophylactic treatment in Colombia: a retrospective non-interventional study".**

**Introduction:**

- Line 72; Hemophilia A and B are rare diseases worldwide and not only in Colombia.
- Line 99: Provider needs to be replaced by Institution

**Materials and methods**:

- Line 110: Does this statement mean that authors stop following patients when they develop inhibitors? If Yes, Why they did not follow them?
- Line 133: Authors need to clarify what they mean by trauma as high percentage of patients had trauma (68%, Table 4).

**Results:**

- Line 173: the statement needs to be changed as mentioned in the comments.
- The titles of most Tables need to be changed (the suggested titles are mentioned on the manuscript).

- Table 4: This table needs to be reviewed and many details can be mentioned in the text instead, e.g. types of bleeding, trauma and surgeries.
- Line 231: Bivariate analysis; This is very important, it needs to be put in a Table, and only the important findings can be written in the text

**Discussion:**

- Line 312: Reference 16 needs to be added.

**Regards**
