# Supplementary material for: The frequency of complications in a cohort of patients diagnosed with hemophilia A and hemophilia B receiving prophylactic treatment in Colombia: A retrospective noninterventional study
Source: PLoS One. 2023 Nov 27;18(11):e0286187. doi: 10.1371/journal.pone.0286187 (PMC10681305; doi:10.1371/journal.pone.0286187)
Supplement: Attachment — Submitted filename: Response to editor comments.docx [file pone.0286187.s003.docx]

Reference ID: PONE-D-22-11504

Title: **The frequency of complications in a cohort of patients diagnosed with hemophilia A and hemophilia B receiving prophylactic treatment in Colombia: a retrospective noninterventional study**

Dear editors

**PLoS One**

We responded to each of the comments made by the evaluators. We are sure that they improve the quality of the manuscript.

1. Thank you for providing the following Funding Statement:

” yes: Reyes JM, Lourdes M and Castano N are paid employees of Pfizer Colombia. Juan David Wilches-Gutierrez, Diana Rocio Arias-Osorio are paid employees of IPS-Especializada. Andres Gaviria-Mendoza, Natalia Castaño-Gamboa, Luis Fernando Valladales-Restrepo, Manuel E. Machado-Duque and Jorge Machado-Alba are paid employees of Audifarma SA. Harrison David Ospina-Arzuaga do not have any conflict of interest.”

We note that one or more of the authors is affiliated with the funding organization, indicating the funder may have had some role in the design, data collection, analysis or preparation of your manuscript for publication; in other words, the funder played an indirect role through the participation of the co-authors.

If the funding organization did not play a role in the study design, data collection and analysis, decision to publish, or preparation of the manuscript and only provided financial support in the form of authors' salaries and/or research materials, please review your statements relating to the author contributions, and ensure you have specifically and accurately indicated the role(s) that these authors had in your study in the Author Contributions section of the online submission form. Please make any necessary amendments directly within this section of the online submission form.  Please also update your Funding Statement to include the following statement: “The funder provided support in the form of salaries for authors [insert relevant initials], but did not have any additional role in the study design, data collection and analysis, decision to publish, or preparation of the manuscript. The specific roles of these authors are articulated in the ‘author contributions’
section.”

If the funding organization did have an additional role, please state and explain that role within your Funding Statement.

Please also provide an updated Competing Interests Statement declaring this commercial affiliation along with any other relevant declarations relating to employment, consultancy, patents, products in development, or marketed products, etc.

**Response: We have updated the statements the conflict of interest, funding and author contributions**

**The authors**
